# Heterogeneity in the prevalence of subclinical malaria, other co-infections and anemia among pregnant women in rural areas of Myanmar: a community-based longitudinal study

**DOI:** 10.1186/s41182-024-00577-5

**Published:** 2024-03-08

**Authors:** Poe Poe Aung, Kay Thwe Han, Wim Groot, Regien Biesma, Zaw Win Thein, Thura Htay, Zaw Lin, Kyin Hla Aye, Matthew Adams, Milena Pavlova

**Affiliations:** 1 Department of Health Services Research, CAPHRI, Maastricht University Medical Center, Faculty of Health, Medicine and Life Sciences, Maastricht University, Maastricht, The Netherlands; 2 Malaria Research Program, Center for Vaccine Development and Global Health, University of Maryland School of Medicine, Baltimore, MD USA; 3 Malaria Consortium, Bangkok, Thailand; 4 Department of Medical Research, Ministry of Health, Yangon, Myanmar; 5 Global Health Unit, Department of Health Sciences, University Medical Center Groningen, Groningen, The Netherlands; 6 National Malaria Control Program, Ministry of Health, Mandalay, Myanmar

**Keywords:** Anemia and co-infections, Longitudinal study, Myanmar, Pregnant women, Subclinical malaria

## Abstract

**Background:**

Due to the low prevalence of clinically suspected malaria among pregnant women in Myanmar, little is known about its impact on mothers and newborns. Helminth and Human Immuno-deficiency Virus (HIV) co-infections cause anemia in pregnant women. This study assessed the prevalence of subclinical malaria and co-infections among pregnant women, and its association with adverse outcomes of pregnancy in the presence of infection.

**Methods:**

A prospective longitudinal study was conducted in 12 villages in two townships in Myanmar between 2013 to 2015. A total of 752 pregnant women, with a mean age of 27 years, were enrolled and followed up once a month until six weeks after childbirth. Prevalence ratio was calculated in the multivariable analysis.

**Results:**

The prevalence of subclinical malaria as measured by nested PCR was 5.7% for either *P. falciparum* or *P. vivax*, 2.7% prevalence of *P. falciparum* and 2.8% prevalence of *P. vivax.* Helminth infections were prevalent in 17% of women, and one woman with an HIV infection was found in our study. The burden of anemia was high, with an overall prevalence of 37% with or without helminth infection, 42% of the women were malaria positive and 43% had dual infections (both malaria and helminth). Only 11 abnormal pregnancy outcomes (7 stillbirths, 2 premature, 2 twins) were identified. Poisson regression showed that women in their first trimester had a 2.9 times higher rate of subclinical malaria compared to women in the third trimester (PR:2.9, 95%CI 1.19, 7.31, *p* = 0.019), women who were enrolled during the wet season were 2.5 times more likely to be malaria positive than the women enrolled in the dry season (PR: 2.5, 95%CI 1.27, 4.88, *p* = 0.008), and the malaria positivity rate decreased by 5% when increased in one year of woman’s age (PR:0.95, 95%CI 0.91, 0.99, *p* = 0.02). In the multivariable regression, the age of respondents was the only significant factor associated with subclinical malaria in pregnancy.

**Conclusions:**

A comprehensive approach of integrating interventions for malaria, anemia, and helminths should be delivered during antenatal care services for pregnant women in rural areas of Myanmar.

**Supplementary Information:**

The online version contains supplementary material available at 10.1186/s41182-024-00577-5.

## Background

Globally, in 2020, 11.6 million (34%) pregnant women experienced malaria during pregnancy, mainly in moderate-to-high transmission settings. However, the total burden of subclinical malaria among pregnant women is unknown [1]. Asymptomatic malaria in pregnancy contributes to low birth weight (LBW), premature birth, babies who are small for gestational age (SGA), and maternal and newborn anemia [2–8]. The burden of clinically suspected malaria in pregnant women in Myanmar was 1–2% in 2016, whereas the prevalence of confirmed positive rapid diagnosis test (RDT) malaria in general populations was 0.23% [9, 10]. The clinical and epidemiologic impacts of treating or not treating these cases are a topic of debate [11]. Treatment is not recommended for asymptomatic parasitemia in low transmission settings, but more evidence is needed to understand the impact of subclinical malaria on pregnancy and newborns [2, 12].

Malaria during pregnancy poses a significant risk of anemia, with its adverse effects extending to newborns via umbilical cord transmission, particularly in expectant mothers with confirmed malaria through microscopy [13–15]. According to the World Health Organization (WHO) in Africa malaria-associated maternal illness and anemia, along with preterm birth and LBW in infants, are predominantly linked to *P. falciparum* infection. In regions with moderate-to-high malaria transmission settings, *P. vivax* infection is associated with maternal anemia and placental parasitemia, which can cause LBW contributing to infant mortality as a result of chronic anemia [16].

Several studies have identified adverse effects of asymptomatic malaria throughout pregnancy. In Colombia, mild-to-moderate malaria-related anemia was found in most of the affected women [17], while in Papua New Guinea chronic placental malaria was significantly associated with LBW and preterm birth in [7]. In India, the prevalence of malaria was 29.3%, with 20.8% being asymptomatic, associated with 92.4% of anemia among pregnant women [18]. A study among antenatal clinics along the Thai–Myanmar border revealed that both *Plasmodium falciparum* and *P. vivax* malaria were associated with preterm birth [4, 19].

Intestinal parasitic infections also increase anemia in pregnant women, resulting in lower pregnancy weight gain and intrauterine growth retardation, leading to LBW [18]. Intestinal parasites with or without malaria co-infection are a major public health problem due to the socio-economic development in low- and middle-income countries, especially in sub-Saharan Africa [20, 21]. Prevalence of soil-transmitted intestinal helminth (STH) with malaria co-infection was 7% in Ethiopia [21] and 16.6% in Ghana [22]. A meta-analysis of findings for African countries revealed that pregnant women infected with hookworm had a 1.3 times higher risk of getting a malaria infection compared with those without hookworm infection [23] and were more susceptible to developing clinical malaria [24, 25].

Studies in sub-Saharan Africa have shown that maternal malaria can influence and increase the risk of Human Immuno-deficiency Virus (HIV) prevalence, mother-to-child HIV transmission through placental malaria, and transient and repeated elevation of HIV viral load for mothers with HIV [26–31]. In low- and middle-income countries, the superimpose of other co-infections, such as HIV and helminths, on malaria in pregnancy causes an increased risk of adverse maternal and perinatal outcomes [5, 26–28, 30, 31]. Studies in Uganda revealed that the dual infection of malaria and intestinal helminths were more common in younger women with HIV co-infection [32, 33]. Instead, malaria and HIV co-infection had a stronger association with anemia compared to helminth infection [32, 33].

There is a substantial knowledge gap concerning malaria in pregnancy, with most observations coming from sub-Saharan African populations. The data available from the South-East Asia region, where transmission patterns differ and mixed infections are prevalent, remain limited. In Myanmar, studies conducted along the Thailand–Myanmar border utilized microscopy to assess malaria in pregnant individuals [4, 19]. However, information regarding subclinical malaria among pregnant women is notably absent. Longitudinal surveillance in the Chittagong Hill Districts of Bangladesh, adjacent to the Myanmar border, revealed that 2.3% of pregnant women harbored asymptomatic malaria, demonstrating 5.4 times higher infection odds compared to men and non-pregnant women. Unfortunately, this study did not measure co-infections with helminths or HIV [34]. Another study from the Thai–Myanmar border highlighted the association between hookworm infection, low birth weight, heightened malaria risk, and maternal anemia [35]. Many studies have looked at the burden of malaria during pregnancy and its effect on maternal health and childbirth, but data did not cover the integration of malaria with other co-infections, such as HIV and intestinal parasites in different transmission endemicity sites in Myanmar [19, 36–38]. More evidence on this problem could help reduce maternal morbidity and mortality, ultimately improving child health outcomes in Myanmar.

This longitudinal study aimed to identify: (1) the prevalence of subclinical malaria among pregnant women; (2) the prevalence of co-infections with HIV and intestinal parasites during pregnancy; (3) the burden of anemia during pregnancy; and (4) the outcome of pregnancy in the presence of infections.

## Methods

### Study design and settings

**Fig. 1:**
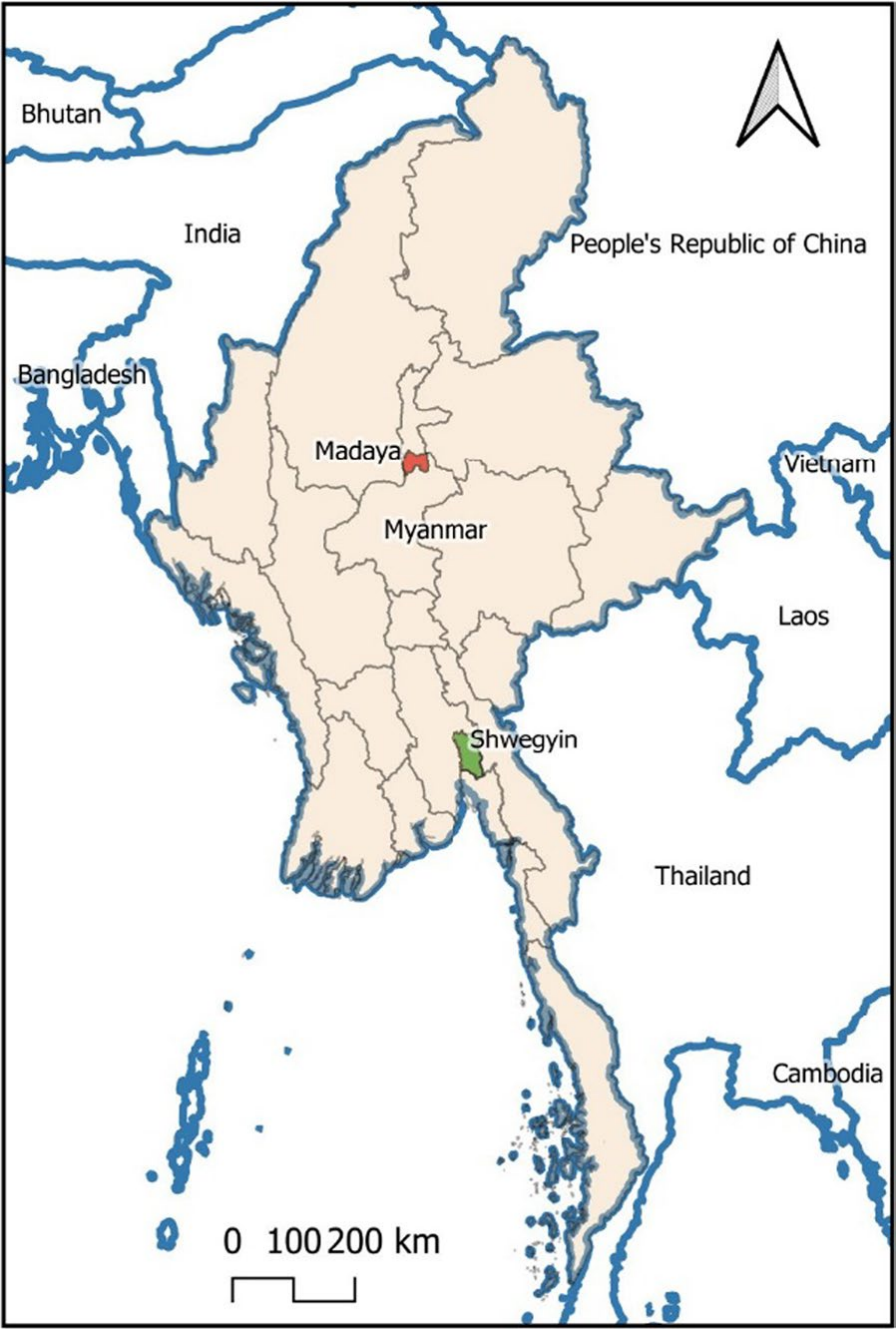
Map of Myanmar with study sites; Shwe Kyin in Bago Region and Madaya in Mandalay Region

The study was designed as a prospective longitudinal study among pregnant women in Myanmar and conducted from August 2013 to March 2015 in 12 malaria-endemic remote villages from two townships, Shwe Kyin and Madaya (six villages from each township), located in Bago and Mandalay Region, respectively (2 of 14 administratively divided regions of Myanmar) (Fig. 1).

Malaria is highly seasonal, with a high positivity rate in the rainy/monsoon season and low in the dry season. The peak rainy season is typically June–September and August–October in Bago and Mandalay Region, respectively [39]. Therefore, data were collected during both rainy and dry seasons.

In Myanmar, midwives are the front-line service providers as well as the local public health workforce embedded within the communities of the villages in the rural settings under the Township Health Department. Midwives are responsible for skilled birth attendance, providing care and treatment of pregnant women, newborn care, not limited to vaccination and primary health care for other infectious diseases, including malaria. The Maternal and Reproductive Health (MRH) program trains and supervises midwives for maternal and child health care. The National Malaria Control Program (NMCP) assigns responsibility for the prevention, diagnosis and treatment of malaria, by supporting rapid diagnostics tests (RDT) and related commodities to midwives, following the Guidelines for Malaria Diagnosis and Treatment in Myanmar [40]. In addition, the Prevention and Treatment of Mother-to-Child Transmission (PMTCT) program is responsible for diagnosis, care and treatment of HIV in pregnant women under the umbrella of the National AIDS Program (NAP).

The Department of Medical Research (DMR) and the NMCP under the Ministry of Health in Myanmar co-led the fieldwork for this study. Thus, this study demonstrated the collaborative effort of different departments, with the integration of three national programs through midwives to detect malaria, anemia, STH and HIV in rural settings in Myanmar.

### Sampling and sample size

We aimed to enroll a sample of approximately 350–400 pregnant women per study site. This sample size was estimated to provide adequate statistical power if the prevalence was at least 10% subclinical malaria by PCR, with a 5% type 1 error. Study participants were recruited primarily through the local midwife at the time of their antenatal care visits. The cohort of pregnant women was recruited with eligibility criteria as follows: (1) confirmed pregnancy, (2) residence within the study villages, (3) the ability and willingness to continue receiving antenatal care at the sub-centers in the study villages, (4) the ability and willingness to comply with the study protocol including monthly follow-up visits until childbirth. Participants were provided with written informed consent. The consenting process comprised pre- and post-test counseling for HIV testing following the HIV testing and treatment guidelines from PMTCT/NAP. Women with HIV reactive had to be referred to a PMTCT/NAP clinic at the township or regional hospital for confirmatory testing to confirm their HIV status as per the national HIV testing algorithm and further care and treatment [41]. Those who had been treated for malaria infections within 4 weeks prior to enrollment and with any clinical illness requiring immediate medical attention were excluded. The latter were referred to appropriate antenatal care services at the study sites.

### Data and biological sample collection

The data and biological samples were collected by trained experienced study midwives from selected villages. Socio-demographic data were collected using a standardized questionnaire including age, education, occupation, gestation of current pregnancy (estimated from the date of last menstrual period, if available, and the measured fundal height), medical history including any chronic medical illness, hospitalizations in the three months prior to enrollment, social history, and review of any clinical symptoms at the time of enrollment. Oral temperature and vital signs were taken, and an abdominal examination was performed to record fundal height and fetal heart rate at enrollment and monthly follow-up visits.

Finger prick blood was taken at enrollment and during monthly follow-up visits to prepare a malaria blood smear and dried blood spot (DBS) for polymerase chain reaction (PCR) analysis. Finger prick blood was also collected at enrollment for the real-time measurement of hemoglobin concentration, using standard hemoglobin point-of-care testing with the Haemocue device (HemoCue 201plus System) and HIV testing using a rapid diagnostic test (SD BIOLINE HIV-1/2 3.0 test).

A stool sample was taken once during the study as soon as it was available before the participant received standard presumptive treatment of STH with mebendazole, following the National Guideline for Antenatal Care for Service Providers [42]. Macro- and microscopic examination of the stool sample was performed in real-time in a local clinical lab by two independent, experienced microscopists.

Due to the limited availability of malaria rapid diagnostic tests at the time of the study, RDT (Standard Diagnostics, Inc., Suwon, Korea) tests were recommended only for those with symptoms suggestive of malaria as defined by the NMCP. Those with acute malaria illness confirmed by RDT or microscopy, stool-positive STH, HIV-positive, or hemoglobin below 10 mg/dL were referred to an antenatal care team for appropriate treatment following the national standard treatment guidelines. At childbirth, the term of pregnancy and pregnancy outcomes, including the newborn’s birth weight, were recorded. Finger prick blood from the mother was taken for malaria smear and PCR analysis at the time of childbirth.

### Laboratory methods

#### Malaria microscopy

Thick- and thin-blood smears were prepared using Giemsa stain and independently examined under a light microscope by two experienced microscopists. One hundred fields of a thick smear were screened before reporting. If the thick smear was positive, 200 fields of a thin smear were screened for species identification. Standard Operations Procedure was developed for internal quality assurance of malaria microscopy according to the WHO Malaria Microscopy Quality Assurance Manual—version 2 [43]. Ten per cent of randomly selected negative slides and all positive slides examined by the field microscopist were cross-checked by an independent microscopist from DMR. All microscopists were certificated as level 1 by WHO monitors.

#### Conventional nested real-time PCR (nested-PCR)

We used nested-PCR as the gold standard laboratory method to identify subclinical malaria [44]. DNA was extracted from dried blood spots using the QIAamp 96 DNA Blood Kit (Qiagen, Valencia, CA) following the manufacturer’s instructions, and the total elution DNA volume for each sample was 100 μL. The nested-PCR was based on primers targeted to the 18S ribosomal RNA gene described previously. Briefly, the first amplification reaction used 2 µL of individual DNA in a 20-µL reaction mixture (0.25 mM dNTP, 10 mM Tris–HCL, 30 mM KCl, 1.5 mM MgCl2, and 1.0 unit of Taq polymerase containing 0.02 mM primers). The second amplification was accomplished by using 2 µL of the first PCR product as a template under the same 20 µL reaction mixture conditions. The cycling conditions were as follows: 95 °C for 5 min, 30 cycles of 95 °C for 30 s, 55 °C for 1 min, and 72 °C for 2 min, followed by a single 60 °C elongation step for 10 min. Amplified products were visualized in 2% agarose gels stained with ethidium bromide. The expected sizes of the *P. falciparum* and *P. vivax* PCR products were 206 and 121 base pairs, respectively.

#### Stool examination

Stool samples were processed and examined within 6 h of collection. Routine microscopic examination was performed to determine the presence of eggs or, *Ascaris lumbricoides*,* Trichuris trichiura* or hookworm using the application of the Kato–Katz method following the WHO guidelines [45].

### Data management

Survey data collected by the trained study midwives were double-entered and validated by experienced data entry staff from DMR using EpiData Entry software (version 3.1 EpiData Association, Odense, Denmark). Validated data were cleaned and analyzed using STATA statistical software (version 15.0, College Station, Texas, USA).

### Measures

*Subclinical or asymptomatic malaria* was defined as positive malaria by microscopy or PCR, without signs or symptoms specific to malaria (fever or history of fever 24 h prior to enrollment) [46, 47]. Malaria infection was counted as mono- or mixed infection of *P. falciparum* or *P. vivax*, detected by microscopy or PCR. Malaria prevalence was calculated as the proportion of tested samples that were positive by each method of detection.

*The age of pregnancy* was defined as the first (1–12 weeks of gestation by fundal height), second (13–26 weeks) and third (27–40 weeks) trimester.

*Anemia* was defined as mild, moderate or severe anemia with hemoglobin concentrations of 10–10.9 g/dL, 7–9.9 g/dL, or less than 7 g/dL, respectively.

*Seasonal variation* was classified by the known seasonal transmission patterns at the time of study enrollment: June–September and August–October were defined as the high transmission season in Shwe Kyin and Madaya sites, respectively, and the rest of the year was defined as low transmission.

### Data analysis

Descriptive analysis was performed using standard measures of variabilities such as the mean, median, and range, or frequencies and percentages, based on the type of variables. The socio-demographic and risk factors between the two study sites were compared using a Chi-square test or Fisher’s exact test. The prevalence of malaria by the two detection methods and the prevalence of anemia in the presence of malaria and/or helminth infection were compared using 2X2 table. Poisson regression with robust variance was used to analyze the prevalence ratio of asymptomatic malaria and identify the association of covariates (age of participant, age of pregnancy, gravidity, presence of clinical symptoms and fever or history of fever in 24 h prior to enrollment, education, occupation, the time of study enrollment (rainy vs dry season), and study site). Risk factors with *p* ≤ 0.2 were considered for the multivariable model, and goodness-of-fist was tested for the best-fit model. The level of significance was set at *p* ≤ 0.05.

## Results

We recruited a total of 752 pregnant women (384 in Shwe Kyin and 368 in Madaya) within two consecutive years. The response rate was 95% at the time of enrollment, and 26% had dropped out by the end of the study.

### Demographic characteristics

Out of 752 pregnant women enrolled in the study, an almost equal number of pregnant women were from the Shwe Kyin and Madaya townships (384 and 368 women) (see Table 1). The pregnant women from both study sites were relatively young (mean age 27 years, standard deviation 6.2), and more than half of the women passed primary school education (52.9% and 56%, respectively) in Shwe Kyin and Madaya. Many pregnant women were primigravid (35.9% in Shwe Kyin and 43.5% in Madaya). Regarding malaria symptoms, most of the women in Shwe Kyin were completely asymptomatic compared to Madaya (97.1% vs 70.9%), but more women from Shwe Kyin had fever at the time of examination compared to Madaya (5.2% vs 0.8%). The mean hemoglobin values of pregnant women in Shwe Kyin and Madaya were similar (10.2 g/dl and 10.4 g/dl, respectively).

**Table 1:** Demographic characteristics of pregnant women at the time of enrollment

Demographic characteristics	Study sites		Total *n* (%)
	Shwe Kyin *n* (%)	Madaya *n* (%)	
	(N = 384)	(N = 368)	(N = 752)
Mean age (SD) (years)	27.2 (6.24)	27.4 (6.23)	27.3 (6.23)
Education			
Illiterate	5 (1.3)	15 (4.1)	20 (2.7)
Can read/write/primary school	203 (52.9)	206 (56.0)	409 (54.4)
With at least secondary school	155 (40.4)	142 (38.6)	297 (39.5)
Gestation (week) (by fundal height)			
Median (IQR) 1st trimester^a^	12 (5–13)	8 (3–13)	10 (3–13)*
Median (IQR) 2nd trimester^b^	22 (14–26)	20 (14–26)	20 (14–26)*
Gravidity			
Primigravid	138 (35.9)	160 (43.5)	298 (39.6)
Secundigravid	89 (23.2)	104 (28.3)	193 (25.7)
Completely asymptomatic	373 (97.1)	261 (70.9)	634 (84.3)^
With fever^c^	20 (5.2)	3 (0.8)	23 (3.1)^
Mean (SD) body weight (Kg)	52.4 (7.50)	51.4 (8.75)	51.9 (8.14)
Mean (SD) hemoglobin (g/dl)	10.2 (1.28)	10.4 (1.31)	10.3 (1.30)*

IQR: interquartile range

^*^p < 0.05 by t-test, ^*p* < 0.05 by Pearson Chi2

^a^Gestational age 1–13 week

^b^Gestational age 14–26 week

^c^Fever defined as axillary temperature ≥ 37.5 °C or reported fever in 24 h prior

### Proportion of subclinical malaria and its seasonal pattern

The prevalence of mixed *P. falciparum* and *P. vivax* malaria was tested by microscopy and nested PCR (nested-PCR) among 752 pregnant women. The prevalence of subclinical malaria by nested PCR was 5.7% for any species, *2.7% for P. falciparum and 2.8% for P. vivax* (see Table 2).

**Table 2:** Prevalence of subclinical malaria among pregnant women in two study sites using microscopy and rt-PCR during longitudinal visits

Pathogen positive	Diagnostic method	Positive rate by study sites		Total* n (%)
		Shwe Kyin *n* (%)	Madaya *n* (%)	
		(*N* = 384)	(*N* = 368)	(*N* = 752)
Any malaria positive	Prevalence by rt-PCR	26 (6.77)	17 (4.62)	43 (5.72)
	Once by microscopy	5 (1.30)	2 (0.54)	7 (0.93)
	Once by rt-PCR	20 (5.21)	12 (3.26)	32 (4.26)
	≥ Twice by microscopy	1 (0.26)	–	1 (0.13)
	≥ Twice by rt-PCR	6 (1.56)	5 (1.36)	11 (1.46)
*P. falciparum* positive	Prevalence by rt-PCR	13 (3.39)	7 (1.90)	20 (2.66)
	Once by microscopy	3 (0.78)	1 (0.27)	4 (0.53)
	Once by rt-PCR	11 (2.86)	6 (1.63)	17 (2.26)
	≥ Twice by microscopy	–	–	–
	≥ Twice by rt-PCR	2 (0.52)	1 (0.27)	3 (0.40)
*P. vivax* positive	Prevalence by rt-PCR	13 (3.39)	8 (2.17)	21 (2.79)
	Once by microscopy	2 (0.52)	1 (0.27)	3 (0.40)
	Once by rt-PCR	9 (2.34)	4 (1.09)	13 (1.73)
	≥ Twice by microscopy	1 (0.26)	–	1 (0.13)
	≥ Twice by rt-PCR	4 (1.04)	4 (1.09)	8 (1.06)
Mixed infection positive**	Prevalence by rt-PCR	–	2 (0.54)	2 (0.27)
	Once by microscopy	–	–	–
	Once by rt-PCR	–	–	–
	> twice by microscopy	–	–	–
	> twice by rt-PCR	–	2 (0.54)	2 (0.27)

^*^Pearson Chi2 and Fisher’s exact test are statistically not significant

^**^P. falciparum and *P. vivax* were tested positive in different time points in monthly visits

The seasonal pattern of malaria positivity by month in the two study townships during the study period (2013–2015) is described in Additional file 1. Overall, malaria positivity in the two study sites was less than 1%, with the lowest of 0.1% in the dry season (February to May) and the highest of 0.5% in the wet season. The malaria positivity pattern comprised two peaks, June–October and December–January, with a noticeable increase of about 0.4–0.5% in September–October in both study sites. The episode of subclinical malaria among pregnant women in two study sites using rt-PCR during longitudinal visits is described in Additional file 2.

### Progression of symptomatic disease

We identified only one pregnant woman who had baseline *P. falciparum* malaria positive by nested PCR, which appeared to be persistent *P. falciparum* malaria positive by microscopy during follow-up. She was 23 years old, secundigravida from Shwe Kyin Township, having symptoms during the baseline visit (temperature = 37.7 °C) but no fever during follow-up visits. She had mild anemia (hemoglobin = 10.6 g/dl), no co-infections by HIV and soil-transmitted helminths, and her pregnancy outcome was normal live birth.

### Proportion of co-infections among pregnant women

The overall prevalence of helminth infection was 10.4% among pregnant women, with the prevalence among women in Shwe Kyin being suggestively higher than the prevalence among women in Madaya (17.8% vs. 2.5%) (see Table 3). Only one woman with HIV was found in Shwe Kyin. The prevalence of subclinical malaria with helminth infection was higher in the Shwe Kyin Township (5.8% vs. 4.6%). Malaria and soil-transmitted infections in pregnant women by villages in two study townships are described in Additional file 3. No co-infection of subclinical malaria with HIV was found in this study.

**Table 3:** Prevalence of co-infections among pregnant women

Prevalence of co-infection among pregnant women	Study sites		Total* n (%)
	Shwe Kyin *n* (%)	Madaya *n* (%)	
Prevalence of HIV infection	**(*N* = 384)**	**(*N* = 368)**	**(*N* = 752)**
	1 (0.26)	0 (0.00)	1 (0.13)
Prevalence of helminth infection	**(*N* = 375)**	**(*N* = 78)**	**(*N* = 453)**
	69 (18.40)	9 (11.54)	78 (17.22)
Prevalence of subclinical malaria with helminth infection	**(*N* = 69)**	**(*N* = 9)**	**(*N* = 78)**
	4 (5.80)	3 (33.33)	7 (8.97)
Prevalence of subclinical malaria with HIV infection	**(*N* = 1)**	**(*N* = 0)**	**(*N* = 1)**
	0 (0.0)	0 (0.0)	0 (0.0)

^*^Pearson Chi2 and Fisher’s exact test are statistically not significant

### Anemia in pregnancy

The proportion of pregnant women with anemia (hemoglobin < 10 g/dl) with or without malaria and soil-transmitted helminths co-infections (see Table 4). More than one-third of pregnant women had anemia (36%) with a similar pattern in both study sites, whether with or without subclinical malaria. Pregnant women who were only malaria positive were found to have a high prevalence of anemia (41.9%) compared to malaria-negative women (36.4%%).

**Table 4:** Prevalence of anemia (hemoglobin ≤ 10 g/dl) in pregnant women with or without co-infections

Prevalence of anemia in pregnant women	Study sites		Total* n (%)
	Shwe Kyin *n* (%)	Madaya *n* (%)	
Prevalence of anemia among pregnant women^**a**^	**(*N* = 384)**	**(*N* = 368)**	**(*N* = 752)**
	152 (39.6)	124 (33.7)	276 (36.7)
Prevalence of anemia without subclinical malaria	**(*N* = 358)**	**(*N* = 351)**	**(*N* = 709)**
	140 (39.1)	118 (33.6)	258 (36.4)
Prevalence of anemia with subclinical malaria infection	**(*N* = 26)**	**(*N* = 17)**	**(*N* = 43)**
	12 (46.2)	6 (35.3)	18 (41.9%)
Prevalence of anemia with helminth infection	**(*N* = 69)**	**(*N* = 9)**	**(*N* = 78)**
	25 (36.2)	3 (33.3)	28 (35.9)
Prevalence of anemia with both infections (subclinical malaria and helminth infection)	**(*N* = 4)**	**(*N* = 3)**	**(*N* = 7)**
	2 (50.0)	1 (33.3)	3 (42.9)

^*^Pearson Chi2 and Fisher’s exact test are statistically not significant

^a^Hemoglobin ≤ 10 g/dL with moderate-severe anemia

The mean hemoglobin level of women with a single infection (“only malaria positive” or “only any stool positive” or “only protozoa positive” or “only helminth positive”) was not much different from those women who had no malaria nor parasitic infections (“both negative”) (see Additional file 4). However, 10 women with multiple parasitic infections only (Additional file 4, Fig. 2d) had a lower hemoglobin level of 9.3 g/dl compared to women with a single infection, either malaria only or any parasitic infection only. In contrast, two women with malaria and protozoa infections (Additional file 4, Fig. 2b) had the lowest hemoglobin level of 8.15 g/dl, among others.

**Fig. 2:**
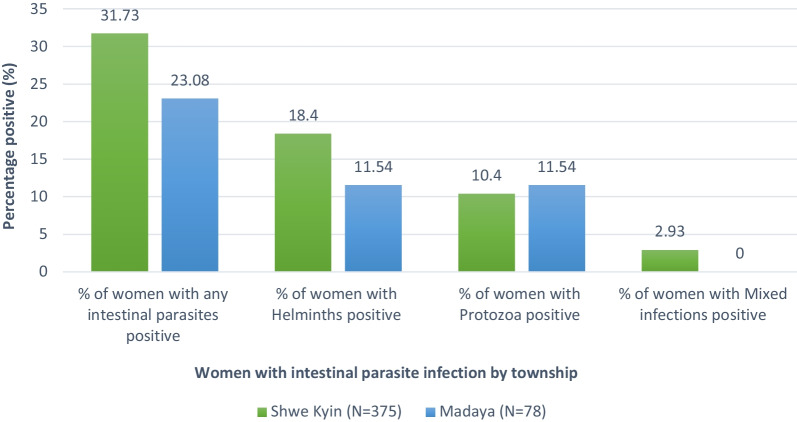
Frequency of observed intestinal parasites in pregnant women by study townships (Pearson’s Chi-squared test *p*-value 0.13)

### Proportion of soil-transmitted infections

The number of stool samples collected and examined from Shwe Kyin was five times larger than those collected in Madaya (375 vs. 78, respectively) (see Fig. 2). About one-third of women from Shwe Kyin were infected with any parasites positive compared to about one-fourth of women from Madaya. Out of 453 stool samples, 17.2% of those with helminth infection (Shwe Kyin = 18.4%, Madaya = 11.5%) were examined by microscopy for soil-transmitted helminths infection. *Ascaris lumbricoides and Trichuris trichiura* were the most predominant infections among pregnant women in both study sites. Pregnant women from Shwe Kyin suffered a significantly higher rate of helminth infections compared to women from Madaya (*Ascaris Lumbricoides*: 9.73% vs. 2.56%; *Trichuris trichiura*: 6.48% vs. 1.28%). Detailed types of observed intestinal parasites (both helminth and protozoa) are presented in Additional file 5.

### Proportion of HIV infection

Among 752 enrolled women, 624 women were tested for HIV, and only one primigravid woman (0.16%) was HIV positive from Shwe Kyin Township (age 27 years). She was anemic (Hb = 7.2 g/dl) but had no fever (temperature = 35.8 °C) at enrollment. Regarding co-infections, she had no malaria based on both microscopy and nested PCR. However, she tested positive for soil-transmitted helminth infection (*Fasciolopsis buski)* from stool examination.

### Outcome of pregnancy in the presence of infections

Among 655 pregnant women with childbirth data, 644 (98.3%) women had a normal pregnancy outcome with normal live birth, while only 11 (1.7%) women had abnormal pregnancy outcomes such as stillbirth (*n* = 7), premature birth (*n* = 2) and twins (*n* = 2). Among those abnormal pregnancy outcomes, both microscopy and nested PCR test results were malaria positive in only one pregnant woman who had the stillbirth outcome, and stool examination results were protozoa positive in only one pregnant woman who had the pregnancy outcome of twins.

### Factors associated with subclinical malaria among pregnant women

Pregnant women in their first trimester had a 2.9 times higher rate of positive malaria compared to the women in the third trimester (95% CI 1.19, 7.31, *p* = 0.019) (see Table 5). Besides, pregnant women who were enrolled during the wet season were 2.49 times more likely to be malaria positive than the women enrolled in the dry season (95% CI 1.27, 4.87, *p* = 0.008). In addition, the malaria positivity rate decreased by 0.95 for increased in one year of woman’s age. (95% CI 0.91, 0.99, *p* = 0.02). In the multivariable regression, the age of the respondents was the only significant factor associated with subclinical malaria in pregnancy.

**Table 5:** Factors influencing prevalence ratio of subclinical malaria in pregnant women by rt-PCR

Potential risk factors	Total (*N* = 752)	rt-PCR (+) n (%) (*N* = 43)	Unadjusted PR (95% CI)	*p-*value*	Adjusted PR (95% CI)	*p-*value*
Site						
Madaya (Ref)	368	17 (4.6)	1		1	
Shwekyin	384	26 (6.8)	1.078 (0.670, 1.736)	0.757	1.412 (0.743, 2.683)	0.292
Season (≥ 3 month in study)						
Dry (Ref)	226	7 (3.1)	1		1	
Wet	526	36 (6.8)	2.492 (1.274, 4.875)	0.008	1.863 (0.821, 4.228)	0.137
Mean age	27.3	24.8	0.952 (0.912, 0.993)	0.022	0.917 (0.851, 0.987)	0.021
Gravidity						
Multigravida (Ref)	259	11 (4.3)	1		1	
Secundigravida	193	13 (6.7)	2.326 (1.232, 4.392)	0.009	1.137 (0.480, 2.690)	0.771
Primigravid	298	19 (6.4)	1.564 (0.832, 2.941)	0.165	0.718 (0.279, 1.850)	0.493
Fever^ at enrollment						
Absent (Ref)	728	41 (5.6)	1		1	
Present	23	2 (8.7)	2.512 (1.011, 6.245)	0.047	1.273 (0.321, 5.049)	0.731
Gestation from fundal height						
Third trimester (Ref)	129	5 (3.9)	1		1	
Second trimester	460	29 (6.3)	1.870 (0.793, 4.410)	0.153	2.080 (0.737, 5.866)	0.166
First trimester	153	8 (5.2)	2.951 (1.191, 7.311)	0.019	1.862 (0.567, 6.118)	0.306
Education					–	–
Illiterate (Ref)	20	1 (5.0)	1			
Literate	706	40 (5.7)	1.841 (0.256, 13.270)	0.545		

*PR* prevalence rate

^*^Poisson regression model with robust variance using the count episode of malaria positivity, ^Fever with 37.5 degree Celsius, PR: prevalence rate

## Discussion

This study sheds light on the notably low prevalence of subclinical malaria infections among pregnant women residing in rural areas, regardless of whether the areas were in high or low-endemic townships in Myanmar. The overall prevalence of any malaria was 5.7%, with *P. falciparum at* 2.7% and *P. vivax at* 2.8%). Despite the geographical distance, these findings are relatively similar to those in a study conducted in three south-eastern states/regions of Myanmar that measured a subclinical malaria prevalence of 3.2% [48]. However, our study revealed heterogeneity in PCR prevalence for both *P. falciparum* (2.7%) and *P. vivax* (2.8%) across the study sites. This is similar to the findings from the Myanmar Malaria Indicators Survey (MIS), which reported an overall PCR prevalence of *P. falciparum* at 0.74% and *P. vivax at* (0.52% [49].

Furthermore, our research highlighted seasonal variation and differences in malaria trends within the studied townships. Moreover, various townships, especially across different states/regions in Myanmar, showed different seasonal variations, geographies and climates. This indicates the unique epidemiological landscape of each township. This suggests the need for finer-scale tracking technique for very low density parasitemia. Poisson regression analysis revealed that women in the first trimester, those who enrolled during the wet season, and younger women had a higher chance of subclinical malaria infections. Similar patterns have been observed in studies conducted in Bangladesh, Myanmar and Papua New Guinea [7, 34, 48].

In our study, the gold standard screening assay, nested PCR, effectively detected subclinical malaria five times more than smear microscopy. Moreover, no persistent positivity was detected in smear microscopy or RDT, whereas nested PCR could detect persistent positivity throughout the longitudinal monthly follow-up visits. However, only one pregnant woman had progressive, symptomatic malaria from a subclinical case. Findings indicated that routine microscopy or RDT would miss a large portion of subclinical malaria infections. In addition, the use of intermittent preventive treatment in pregnancy (IPTp) is the proven preventive method for malaria in pregnancy in African settings [50–52]. However, in this very low prevalence of subclinical malaria in Myanmar, IPTp is less advantageous as an effective preventive measure. However, the burden of subclinical malaria in Asia should not be neglected, as adverse pregnancy outcomes associated with malaria infection are still a concern. This calls for strengthening the surveillance system to achieve early detection of subclinical malaria, which would likely be beneficial for pregnant women and the general population in general, given climate change and the likely impact on the pattern of malaria infections.

It was found that helminth infection was higher among pregnant women from Shwe Kyin compared to Madaya (18.4% vs. 11.5%). Studies from the Thai–Myanmar border revealed that the occurrence of STH infection in the refugee and migrant populations was 30.8% and 16.7%, respectively, which was comparable to the prevalence in our study [53]. Findings also demonstrated that about one-third (35.9%) of pregnant women who had helminth infections were anemic similar to the other studies [21, 53, 54]. As a significant proportion of pregnant women in this study were infected with helminths, the regular MRH program should be strengthened and integrated into routine antenatal care visits to prevent other associated outcomes, such as anemia, for pregnant women.

Regarding co-infections with malaria, an insignificant number of pregnant women infected by subclinical malaria had helminth infections (0.9%), indicating much lower co-infections compared to a study from Nigeria, which had a significant proportion of pregnant women who were infected with malaria, HIV, and helminth infections, leading to severe anemia [55]. In this study, only one HIV-positive woman was found, which indicated very low HIV positivity among pregnant women in the rural areas of Myanmar. This study might suggest that malaria and HIV co-infection may be uncommon in Myanmar, unlike previous studies suggest [55, 56].

In our study, more than one-third of pregnant women had anemia with or without co-infections. The prevalence of anemia among pregnant women in Myanmar was reported at 47.8% in 2019, according to the World Bank collection of development indicators compiled from officially recognized sources [57]. Compared to these World Bank data, the proportion of pregnancy-related anemia is relatively low in our study. Only about one-third of pregnant women in our study had anemia, and this was considerably high in our study with or without co-infections. Therefore, although we could not conclude that anemia is associated with malaria and/or helminth infections, the proportion of pregnant women having anemia should not be neglected. It is recommended to screen for early diagnosis and treatment of anemia during antenatal visits, and further study should explore the cause of anemia among pregnant women. In addition, the data from this study might underestimate the prevalence of malaria as we recruited and tested pregnant women who sought antenatal care from midwives. These pregnant women might not be representative of all pregnant women in the community. Findings suggested that the MRH program should consider supporting midwife care by providing one-stop testing of hemoglobin levels in rural areas to enhance early diagnosis and treatment of anemia among pregnant women in Myanmar.

We found seven stillbirths and two premature babies as outcomes of pregnancy, although the association with subclinical malaria was not statistically significant. Many studies in Africa and Papua New Guinea show that the risk of LBW and premature birth is doubled if pregnant women had subclinical malaria positive with co-infections [58–61]. In our study, we used a descriptive approach in which it was not possible to evaluate the pregnancy outcome due to the small sample size. Perhaps there is a large population of women who do not see midwives during pregnancy and a very low prevalence of subclinical malaria. Future research is required to determine the outcomes of pregnancy among pregnant women with subclinical malaria.

This is the first longitudinal study to investigate subclinical malaria and co-infections among pregnant women in Myanmar. The other strengths of the study include the successful recruitment and enrollment of pregnant women through local midwives at antenatal visits, the low dropout rate, enhanced follow-up visits and study adherence, responsiveness and participation of the local community, and building community trust in the study sites. This was possible to achieve due to community engagement and multi-sector involvement. There was a significant collaborative effort among the townships’ public health workforce, including midwives. There was also strong support from the township health department in the process of the recruitment and implementation at the frontline from the NMCP and NAP in the training of study teams, as well as administrative and logistics support for testing and treatment of malaria and HIV. Furthermore, the contribution of the Parasitology Research Division from the DMR in training for midwives and laboratory analysis of smear microscopy and nested PCR was remarkable. In addition, this study demonstrated research capacity by strengthening the local public health workforce and assessing the training needs of the study team to efficiently recruit, enroll, and follow the target study population for future clinical studies.

This study also has some limitations. One of the limitations was the convenient sampling of villages and pregnant women through the midwives at the antenatal care visits. It might have led to selection bias, and our sample may not be representative of those women who are not presenting for antenatal care with the midwives. In addition, only one-fifth (21%) of the stool samples from the participants in Madaya were tested due to the lack of a research unit in Madaya. Furthermore, the laboratory technician assigned to stool collection and processing went on maternity leave during the study period without any replacement. This might have led to an underestimation of the prevalence of helminth infection in Madaya Township. Also, the prevalence of subclinical malaria was very small in our study, resulting in the inability to evaluate the pregnancy outcome and effect of co-infection among pregnant women.

### Implications of the study

In rural settings in Myanmar, the three national programs (Maternal and Reproductive Health, NMCP, NAP) should be integrated as a comprehensive one-stop service for pregnant women during their antenatal visits to midwives. This approach could be promising in ensuring early diagnosis and treatment of malaria and anemia, deworming programs, diagnosis and proper referral of women with HIV to tertiary health centers. Further research should use a larger samples size to better identify the risk factors for subclinical malaria and its impact on pregnant women and newborns in low-prevalence settings. In addition, further research designed based on qualitative approach, such as in-depth interviews with the sample group, should be conducted. It will help to better understand the social and cultural context factors that could potentially influence subclinical malaria in pregnant women and to find out possible solutions. Surveillance systems should be strengthened and improved to detect subclinical malaria (without symptoms) to achieve the goal of malaria elimination by 2030.

## Conclusions

This study revealed a low prevalence of diverse subclinical malaria and co-infections among pregnant women in rural areas, spanning both high and low malaria endemicity sites in Myanmar. Despite this, overlooking subclinical malaria in vulnerable populations would be unwise, especially considering the aim for malaria elimination by 2030. Notably, pregnancy-related anemia prevalence remained significantly high across both study sites, irrespective of the presence of subclinical malaria or co-infections. This underscores the urgent need for improved early detection and treatment of anemia among pregnant women in rural areas.

Moreover, our findings indicated the presence of helminth infections among pregnant women, signaling the necessity to continue strengthening the deworming programs. A comprehensive approach targeting pregnant women, integrating interventions for malaria, anemia, and helminths should be advocated through antenatal care services delivered by basic health staff in rural areas of Myanmar. Strengthening these integrated efforts becomes crucial for addressing the health challenges faced by pregnant women in these regions.

### Supplementary Information

**Additional file 1: Table S1.** Malaria and soil-transmitted infections in pregnant women by villages in two study townships.

**Additional file 2: Table S2.** Frequency of subclinical malaria episode among pregnant women in two study sites using rt-PCR during longitudinal visits (line graph).

**Additional file 3: Table S3.** Type of observed intestinal parasites.

**Additional file 4: Figure S1.** Malaria positivity by month in each study site (August 2013 to March 2015).

**Additional file 5: Figure S2.** Hemoglobin concentration in pregnant women with and without co-infections.

## Data Availability

Data are not available in public domain. However, data are available with the corresponding author (PPA) and may be made available upon request at the following e-mail: p.aung@maastrichtuniversity.nl.

## Abbreviations

DBS: Dried blood spot
DMR: Department of Medical Research
HIV: Human Immuno-deficiency Virus
IPTp: Intermittent preventive treatment in pregnancy
LBW: Low birth weight
MIS: Malaria Indicators Survey
MRH: Maternal and Reproductive Health program
NAP: National AIDS Program
NMCP: National Malaria Control Program
PCR: Polymerase chain reaction
PMTCT: Prevention and Treatment of Mother-to-Child Transmission
RDT: Rapid diagnosis test
PCR: Conventional nested real-time PCR
SGA: Small for gestational age
STH: Soil-transmitted intestinal helminth
WHO: World Health Organization
