# Supplementary material for: Heterogeneity in the prevalence of subclinical malaria, other co-infections and anemia among pregnant women in rural areas of Myanmar: a community-based longitudinal study
Source: Trop Med Health. 2024 Mar 8;52:22. doi: 10.1186/s41182-024-00577-5 (PMC10921590; doi:10.1186/s41182-024-00577-5)
Supplement: Supplementary file 1 — Additional file 1: Table S1. Malaria and soil-transmitted infections in pregnant women by villages in two study townships. [file 41182_2024_577_MOESM1_ESM.docx]

Table S1. Malaria and soil-transmitted infections in pregnant women by villages in two study townships

| **Township** | **Village (n)** | **Median (range) DBS/person** | **# (%) rt-PCR data available > once** | **# (%) rt-PCR positive >once** | **# (%) stool samples collected** | **# (%) stool results available** | **# (%) STH positive** | **# (%) Malaria+STH** |
| --- | --- | --- | --- | --- | --- | --- | --- | --- |
| SK | Village A (41) | 4 (1-6) | 41 (100) | 0 | 41 (100) | 41 (100) | 6 (14.63) | 0 |
| SK | Village B (50) | 4 (1-7) | 50 (100) | 8 (16.00) | 52 (104) | 50 (100) | 9 (18.00) | 3 (6.00) |
| SK | Village C (61) | 5 (1-7) | 61 (100) | 3 (4.92) | 56 (91.80) | 56 (91.8) | 14 (22.95) | 1 (1.64) |
| SK | Village D (58) | 5 (1-8) | 58 (100) | 3 (5.17) | 63 (108.62) | 58 (100) | 9 (15.52) | 0 |
| SK | Village E (97) | 4 (1-7) | 97 (100) | 4 (4.12) | 96 (98.97) | 93 (95.88) | 17 (17.53) | 0 |
| SK | Village F (77) | 4 (1-6) | 77 (100) | 8 (10.39) | 93 (120.78) | 77 (100) | 14 (18.18) | 0 |
| Total Shwe Kyin (384) | | 4 (1-8) | 384 (100) | 26 (6.77) | 401 (104.43) | 375 (97.66) | 69 (18.4) | 4 (1.04) |
| MDY | Village G (51) | 3 (1-6) | 51 (100) | 1 (1.96) | 1 (1.96) | 1 (1.96) | 0 | 0 |
| MDY | Village H (99) | 4 (1-9) | 99 (100) | 7 (7.07) | 25 (25.25) | 25 (25.25) | 2 (2.02) | 1 (1.01) |
| MDY | Village I (77) | 6 (1-9) | 77 (100) | 3 (3.90) | 29 (37.66) | 29 (37.66) | 0 | 0 |
| MDY | Village J (57) | 4 (1-8) | 57 (100) | 1 (1.75) | 9 (15.79) | 9 (15.79) | 2 (3.51) | 0 |
| MDY | Village K (65) | 3 (1-7) | 65 (100) | 4 (6.15) | 2 (3.08) | 2 (3.08) | 2 (3.08) | 2 (3.08) |
| MDY | Village L (19) | 6 (3-8) | 19 (100) | 1 (5.26) | 12 (63.16) | 12 (63.16) | 3 (15.79) | 0 |
| Total Madaya (368) | | 4 (1-9) | 368 (100) | 17 (4.62) | 78 (21.20) | 78 (21.20) | 9 (11.54) | 3 (0.82) |
| Grand Total (752) | | 4 (1-9) | 752 (100) | 43 (5.72) | 479 (63.70) | 453 (60.24) | 78 (17.22) | 7 (0.93) |

DBS: Dry blood spot, STH: Soil transmitted helminth infection
