# Supplementary material for: Heterogeneity in the prevalence of subclinical malaria, other co-infections and anemia among pregnant women in rural areas of Myanmar: a community-based longitudinal study
Source: Trop Med Health. 2024 Mar 8;52:22. doi: 10.1186/s41182-024-00577-5 (PMC10921590; doi:10.1186/s41182-024-00577-5)
Supplement: Supplementary file 2 — Additional file 2: Table S2. Frequency of subclinical malaria episode among pregnant women in two study sites using rt-PCR during longitudinal visits (line graph). [file 41182_2024_577_MOESM2_ESM.docx]

Table S2. Frequency of subclinical malaria episode among pregnant women in two study sites using rt-PCR during longitudinal visits

| **Frequency of subclinical malaria episode using rt-PCR** | **Study sites** | | **Total***  **(N=752)**  **n (%)** |
| --- | --- | --- | --- |
|  | **Shwe Kyin (N=384)**  **n (%)** | **Madaya (N=368)**  **n (%)** |  |
| Negative | 358 (93.23) | 351 (95.38) | 709 (94.28) |
| 1 time positive | 20 (5.21) | 10 (2.72) | 30 (3.99) |
| 2 times positive | 3 (0.78) | 3 (0.82) | 6 (0.80) |
| 3 times positive | 2 (0.52) | 1 (0.27) | 3 (0.40) |
| 4 times positive | 1 (0.26) | 2 (0.54) | 3 (0.40) |
| 5 times positive |  | 1 (0.27) | 1 (0.13) |

*Pearson chi2 and Fisher’s exact test are statistically not significant
