# Supplementary material for: Heterogeneity in the prevalence of subclinical malaria, other co-infections and anemia among pregnant women in rural areas of Myanmar: a community-based longitudinal study
Source: Trop Med Health. 2024 Mar 8;52:22. doi: 10.1186/s41182-024-00577-5 (PMC10921590; doi:10.1186/s41182-024-00577-5)
Supplement: Supplementary file 3 — Additional file 3: Table S3. Type of observed intestinal parasites. [file 41182_2024_577_MOESM3_ESM.docx]

Table S3. Type of observed intestinal parasites

| **Intestinal parasites** | **Type of organism** | **Study sites** | | **Total*** **(N=453)**  **n (%)** |
| --- | --- | --- | --- | --- |
|  |  | **Shwe Kyin (N=375)**  **n (%)** | **Madaya (N=78)**  **n (%)** |  |
| Helminths | *Ascaris lumbricoides* | 39 (10.4) | 2 (2.56) | 41 (9.05) |
|  | *Trichuris trichiura* | 26 (6.93) | 1 (1.28) | 27 (5.96) |
|  | *Hookworm eggs* | 10 (2.67) | - | 10 (2.21) |
|  | *Enterobius vermicularis* | 4 (1.07) | 1 (1.28) | 5 (1.1) |
|  | *Schistosoma mekongi* | 2 (0.53) | 3 (3.85) | 5 (1.1) |
|  | *Clonorchis sinensis* | 3 (0.8) | - | 3 (0.66) |
|  | *Fasciolopsis buski* | 3 (0.8) | - | 3 (0.66) |
|  | *Paragonimus westermani* | 2 (0.53) | - | 2 (0.44) |
|  | *Tapeworm spp:* | 2 (0.53) | - | 2 (0.44) |
|  | *Taenia spp. Eggs* | 1 (0.27) | 1 (1.28) | 2 (0.44) |
|  | *Strongyloides stercoralis larva* | 1 (0.27) | 1 (1.28) | 2 (0.44) |
|  | *Diphyllobothrium latum* | 1 (0.27) | 1 (1.28) | 2 (0.44) |
|  | *Hymenolepis nana* | 1 (0.27) | - | 1 (0.22) |
|  | *Fasciola hepatica* | 1 (0.27) | - | 1 (0.22) |
| Total Helminth |  | 96 (25.6) | 10 (12.82) | 106 (23.4) |
| Protozoa | *Blastocystis hominis* | 16 (4.27) | 4 (5.13) | 20 (4.42) |
|  | *Entamoeba histolytica* | 14 (3.73) | 1 (1.28) | 15 (3.31) |
|  | *Balantidium coli* | 7 (1.87) | 1 (1.28) | 8 (1.77) |
|  | *Entamoeba coli* | 6 (1.6) | - | 6 (1.32) |
|  | *Giardia* | 5 (1.33) | 1 (1.28) | 6 (1.32) |
|  | *Giardia lamblia* | 4 (1.07) | 1 (1.28) | 5 (1.1) |
|  | *Trichomonas intestinalis* | 3 (0.8) | - | 3 (0.66) |
|  | *Toxocara canis* | 1 (0.27) | - | 1 (0.22) |
|  | *Giardia intestinalis* | - | 1 (1.28) | 1 (0.22) |
|  | *Toxocara cati* | 1 (0.27) | - | 1 (0.22) |
| Total Protozoa |  | 57 (15.2) | 9 (11.54) | 66 (14.57) |
| Grand total  Intestinal parasites |  | 153 (40.8) | 19 (24.36) | 172 (37.97) |

*Total of 401 and 78 stool samples received from Shwe Kyin and Madaya site, respectively
