# Supplementary material for: Heterogeneity in the prevalence of subclinical malaria, other co-infections and anemia among pregnant women in rural areas of Myanmar: a community-based longitudinal study
Source: Trop Med Health. 2024 Mar 8;52:22. doi: 10.1186/s41182-024-00577-5 (PMC10921590; doi:10.1186/s41182-024-00577-5)
Supplement: Supplementary file 4 — Additional file 4: Figure S1. Malaria positivity by month in each study site (August 2013 to March 2015). [file 41182_2024_577_MOESM4_ESM.docx]

Figure S1. Malaria positivity by month in each study site (August 2013 to March 2015)
