# Supplementary material for: Heterogeneity in the prevalence of subclinical malaria, other co-infections and anemia among pregnant women in rural areas of Myanmar: a community-based longitudinal study
Source: Trop Med Health. 2024 Mar 8;52:22. doi: 10.1186/s41182-024-00577-5 (PMC10921590; doi:10.1186/s41182-024-00577-5)
Supplement: Supplementary file 5 — Additional file 5: Figure S2. Hemoglobin concentration in pregnant women with and without co-infections. [file 41182_2024_577_MOESM5_ESM.docx]

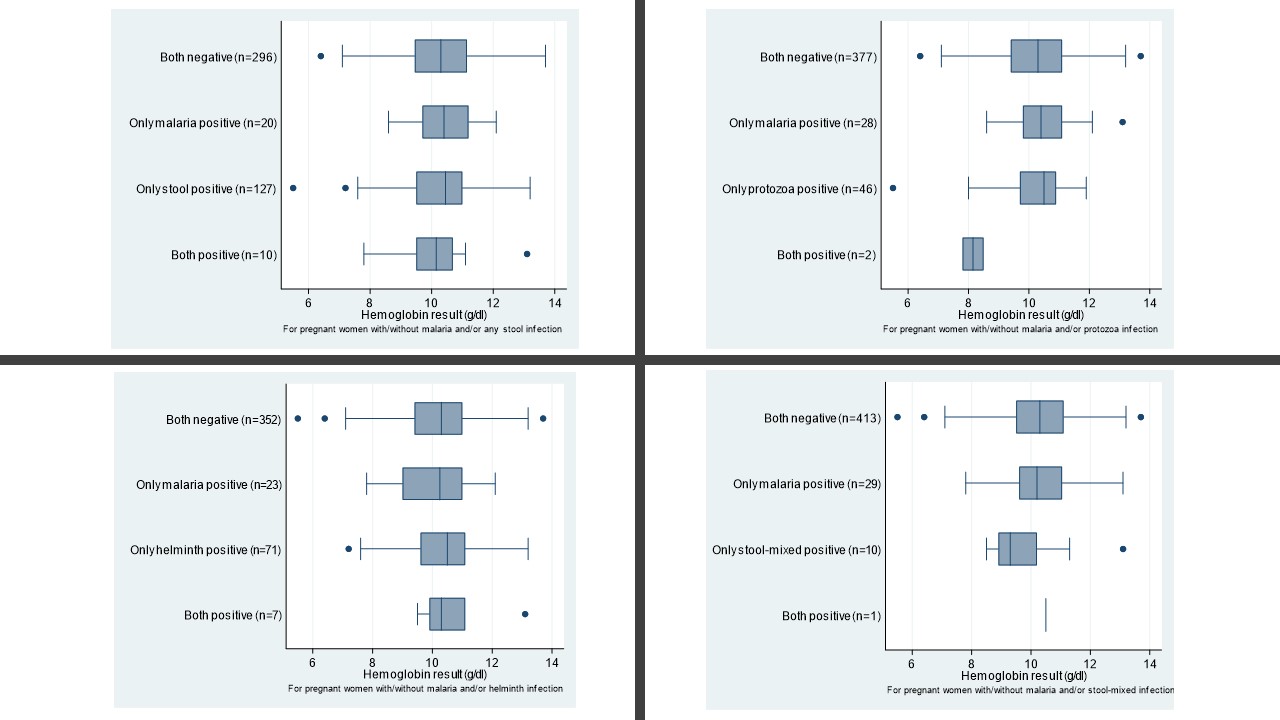


Fig 2b

Fig 2a

Fig 2c

Fig 2d

Figure S2. Hemoglobin concentration in pregnant women with and without co-infections
